# Male sex assignment in severely virilized 46,XX children with congenital adrenal hyperplasia

**DOI:** 10.3389/fendo.2026.1765653

**Published:** 2026-03-02

**Authors:** Nikolett Jusztina Beniczky, Emma Lenke Magyar, Ágnes Sallai, Zoltán Jenővári, Zita Sükösd, Zsolt Vajda, Ya-Lea Steenkamp, Nikolette Szücs, Rita Bertalan

**Affiliations:** 1 Pediatric Center, Semmelweis University, Budapest, Hungary; 2 Department of Toxicology and Metabolism, Heim Pál National Pediatric Institute, Budapest, Hungary; 3 Studer Family Children’s Hospital at Ascension Sacred Heart, Florida State University, Pensacola, FL, United States; 4 Department of Internal Medicine and Oncology, Semmelweis University, Budapest, Hungary

**Keywords:** 46,XX male, congenital adrenal hyperplasia, gender assignment, gender identity, sex assignment, virilization, virilized external genitalia

## Abstract

**Introduction:**

Increased androgen production in 46,XX individuals with congenital adrenal hyperplasia leads to a variable degree of external genital virilization, often complicating the decision regarding sex assignment after birth.

**Methods:**

In this single tertiary center retrospective study, we analyzed individuals with 46,XX karyotype and 21-hydroxylase deficiency with Prader score V or EMS 8-9, who were assigned and reared as male in the last 40 years. In parallel, we conducted a comprehensive review of published case reports on 46,XX male CAH patients to gain insight into their gender identity outcomes, surgical interventions, and quality of life.

**Results:**

We identified four severely virilized 46,XX CAH patients, who were assigned and reared as male and have maintained a stable male gender identity. In addition, we found 63 similar published cases in the literature between 1963 and 2025. Outcome data were available in 50 cases. Among them, 43/50 patients were satisfied with male gender identity, although 3/43 underwent male to female and back to male reassignment.

**Conclusion:**

Our experience with four patients aligns with several published case reports showing that 46,XX CAH infants with severe genital virilization reared as male may develop and maintain a stable male gender identity throughout life. However, if male sex assignment is chosen for a 46,XX child, parents should be counseled about fertility implications, future surgical needs, potential height-related concerns and the possibility of testosterone treatment. Nevertheless, individualized care, shared decision-making, parental involvement and family support appear to be essential for a positive outcome.

## 1 Introduction

The most common cause of genital atypia in newborns with 46,XX karyotype is the classic form of congenital adrenal hyperplasia (CAH) due to 21-hydroxylase deficiency (21OHD) (1, 2). The pathogenic variant of *CYP21A2* gene, which encodes the steroid 21-hydroxylase enzyme, leads to inefficient cortisol biosynthesis and thus increased adrenal androgen excess. The varying degrees of external genital virilization in 46,XX patients develop prenatally during early fetal life (3–5).

The salt wasting (SW) and simple virilizing (SV) forms of classic CAH occur in both sexes, and increased prenatal androgens result in external genital virilization in females (i.e. clitoromegaly, labial fusion, urogenital sinus). The degree of virilization can be assessed by the Prader stages or EMS/EGS (external masculinization score/external genitalia score), the completely virilized external genitalia are defined as Prader stage V or EMS/EGS 9 in cases with no palpable testes (6).

In this rare condition, the decision on sex assignment represents a major challenge, and it should be made in consultation with a team of health care professionals, such as an involved pediatric endocrinologist, psychologist, clinical geneticist and pediatric surgeon, who are experienced in DSD (differences/disorders of sex development) and a fully informed family (7, 8).

Several guidelines and recommendations are available; however, they differ considerably in their approaches to the sex of rearing, reflecting the complexity and heterogeneity of this population. The majority of the recommendations and case studies previously suggested female assignment for 46,XX CAH patients. However, there are case reports of patients with 46,XX karyotype and CAH who have been successfully reared as males. In 2000, the American Academy of Pediatrics (AAP) recommended that these individuals be raised as girls, primarily due to their fertility potential (9). The 2002 Consensus Statement written by LWPES (Lawson Wilkins Pediatric Endocrine Society) and ESPE (European Society for Pediatric Endocrinology) Working Group recommended rearing these patients as females, supplemented by the fact that there is no clear evidence to support even completely virilized (Prader stage V) 46,XX CAH infants’ male assignment (10). The 2018 Endocrine Society Clinical Practice Guideline emphasizes the importance of thorough discussions, particularly concerning the fertility implications of sex assignment (5). According to the North American DSD Clinician Survey, based on pediatric endocrinologists and urologists’ experiences over the past two decades, there is an increasing trend toward recommending a gender “other than girl” for 46,XX CAH patients with severe virilization and to perform genital surgery later in life (11). As no universally optimal approach exists, shared decision-making is essential when determining sex assignment or planning surgical interventions (12–14).

The purpose of our study was to present two of four cases from our center over the past 40 years of phenotypic male, but genotypic female patients with CAH, who were assigned male at birth and reared as males. We aim to retrospectively analyze the challenges they encountered from diagnosis through young adulthood and to assess whether male sex assignment represented a viable choice in these cases. We also summarized previously published case reports of 46,XX CAH patients with initial male sex assignment to provide further insight into outcomes and quality of life.

## 2 Materials and methods

We retrospectively identified patients with CAH, 46,XX karyotype and male sex assignment between 1985 and 2025 at our single tertiary pediatric center. All patients were evaluated by a multidisciplinary team including pediatric endocrinologist, pediatric surgeon and pediatric psychologist. We analyzed their medical records from birth to young adult age. Cytogenetic analysis was performed to determine the karyotype, and the diagnosis was confirmed by molecular genetic analysis. Psychological assessment was performed using the Strengths and Difficulties Questionnaire (SDQ) to evaluate emotional and behavioral problems. In Patients 1 and 2, the SDQ was completed by both the patients and their parents, while in the remaining two cases, the assessment could not be completed due to poor compliance.

In parallel, we collected case reports published up to 2025 through a PubMed search describing 46,XX CAH patients, who were initially assigned male at birth. The search terms included: congenital adrenal hyperplasia; adrenogenital syndrome; 21-hydroxylase deficiency, 11-hydroxylase deficiency; differences (disorders) of sex development; 46,XX CAH; 46,XX males; (male) gender assignment; sex assignment; (male) gender identity; masculinized genitalia; masculinization of external genitalia, virilized genitalia; virilization of external genitalia; ambiguous genitalia/genital ambiguity; gender dysphoria; female pseudohermaphroditism. Titles and abstracts were screened independently by the authors to assess eligibility.

## 3 Results

### 3.1 Case series of our two patients

#### 3.1.1 Patient 1

The first patient was born at term with a birthweight of 3950 grams. Prenatal karyotyping was performed due to increased nuchal fold thickness, which revealed a 46,XX karyotype, although fetal ultrasound showed male-type external genitalia. After delivery, the physical examination was remarkable for bilateral cryptorchidism and Prader stage V genitalia. In the first week of life, the patient had hyponatremia, hyperkalemia and elevated 17-hydroxyprogesterone (17-OHP: 363 nmol/L, RR: 1.5–15 nmol/L), which confirmed the suspected diagnosis: salt-wasting congenital adrenal hyperplasia (SW CAH) (**Table 1**). Glucocorticoid and mineralocorticoid replacement therapy was initiated immediately. Molecular genetic analysis revealed a homozygous mutation of I172N at the *CYP21A2* gene. Abdominal ultrasound showed female internal genitalia (uterus, ovaries, vagina). The decision regarding male sex assignment was made by a multidisciplinary team, including pediatric endocrinologists, surgeons, gynecologists and the well-informed parents together. Based on the decision, partial hysterectomy and bilateral oophorectomy were performed at 20 months of age, and testicular prostheses (3–3 ml) were implanted, as well. His growth and weight curve followed the 50^th^ percentile, and pubarche started at 10.5 years of age. His bone age was advanced by 2 years between the ages of 7 and 13. At 13 years of age, androgen substitution with testosterone depot intramuscular injection was started at a dose of 50mg/4 weeks and increased until the full dose (250 mg/4 weeks). From the age of 11, he suffered from recurrent dysuria. On the physical examination scarred, narrow urethral opening and chordee of the penis were found. Dropping and urethral stenosis with vaginal urination was treated with simple urethral self-dilatations (5mm) and no vaginal removal was needed. The patient had chordee during erection, therefore he underwent chordee repair surgery. The testicular prostheses were replaced with appropriately sized ones at the age of 14.

**Table 1:** Our 46,XX CAH patients with initial male sex assignment.

Patient no.	CAH diagnosis/mutation	Age at diagnosis	Diagnostic findings	Sex assignment	Gender identity	Surgical intervention (age/year if available)	Outcome
Patient 1 year of birth: 2000s	SW CAH CYP21A2 gene homozygous mutation of I172N	3 weeks	Prader V/EMS 9 genitalia, hyponatremia (128–132 mmol/L), hyperkalemia (5.1-6.5 mmol/L), elevated 17-OHP (363 nmol/L; RR: 1.5–15 nmol/L)	male	male	oophorectomy, hysterectomy, testicular prosthesis implantation at 20 months of age, urethral dilatation at 11 years of age, chordee repair surgery	satisfied with male gender at the last medical follow-up (17 years), but having anxiety problems
Patient 2 year of birth: 2000s	SW CAH CYP21A2 gene heterozygous mutations in intron2 splice, Q318X and R356W	1 week	Prader V/EMS 9 genitalia, elevated 17-OHP (396 nmol/L; RR: 1.5–15 nmol/L)	male	male	salpingo-oophorectomy and testicular prosthesis implantation at 3 years of age, chordee repair surgery at 11 years of age	satisfied with male gender at last medical follow-up (17 years)

RR, reference range.

The most challenging aspect of this patient’s care was his anxiety, despite the absence of gender dysphoria. At the age of 16.5 years, his height was 170.5 cm (25-50th percentile, SDS -0,5 according to male percentile chart and SDS + 0,88 according to the female percentile chart). However, since his brother is significantly taller (180 cm), he is constantly worried about his own height. Assessment of Patient 1’s current emotional and behavioral problems using the SDQ revealed significant mental health difficulties. According to the parent-report versions of SDQ emotional symptoms, peer problems and pro-social behavior were found to be problematic, whereas the self–report questionnaire similarly revealed difficulties with peer problems and pro-social behavior domains. Within the family system, there was shame around the DSD diagnosis and the topic was mostly avoided in discussions. It is important to highlight that the patient’s mother suffers from chronic anxiety, which can significantly influence the child’s mental health, even if the male sex assignment was never questioned (15).

#### 3.1.2 Patient 2

Our second patient was born at term with a birthweight of 3500 grams from an uneventful pregnancy with severely virilized external genitalia (Prader stage V, EMS 8) and bilateral cryptorchidism. On the 5^th^ day of life, salt-wasting syndrome manifested with hyponatremia and hyperkalemia; therefore, hormone replacement therapy with hydrocortisone and fludrocortisone was initiated. The laboratory results showed elevated 17-OHP (396 nmol/L, RR: 1.5–15 nmol/L). Based on clinical symptoms and laboratory findings, the diagnosis of SW CAH was given (**Table 1**). The patient was found to have 46,XX karyotype. The molecular genetic examination revealed heterozygous mutations in intron2 splice, Q318X and R356W at the *CYP21A2* gene. Later, we found that the mother is Q318X and the father is intron 2 splice heterozygous carrier. At 19 months of age, laparoscopic exploration showed female internal genitalia, and gonadal biopsy confirmed ovarian structures bilaterally. The decision about sex assignment was a result of shared decision-making process between the medical team and the fully informed parents, who insisted on male gender assignment. According to the decision, bilateral salpingo-oophorectomy was performed, and testicular prostheses (3–3 ml) were implanted at the age of 3 years. Fludrocortisone therapy had to be transiently discontinued due to high blood pressure (150/80 mmHg at 3 years of age). His pubarche started at 9 years of age. At 11 years, he underwent chordee repair surgery. Testosterone replacement therapy was initiated at 13.5 years (testosterone <0.69 nmol/L [RR 13–14 years: ♂ 0.35-12.15 nmol/L, ♀ 0.28-1.7 nmol/L]; LH 73.4 IU/L [RR 13–14 years: ♂ 1–6 IU/L, ♀ 2–8 IU/L]; FSH 111 IU/L [RR 13–14 years: ♂ 1-4.5 IU/L; ♀ 3–8 IU/L]). Patient 2 had good parental support for his male sex assignment and experienced no gender dysphoria. Assessment with the SDQ revealed no mental health difficulties. The parents’ report and self-report versions did not disclose any psychological difficulties, all sub-scales’ scores were in the healthy range.

### 3.2 Overview of the relevant literature and summary of reported cases

Dessens et al. based on an extensive literature review between 1950 and 2005 found that 12% (4/33) of the 46,XX CAH patients assigned as male had serious gender dysphoria, while only 5% (13/250) of the patients raised as females had gender dysphoria. However, the difference was not statistically significant, and no distinction was made between Prader stages of the external genitalia (15). A few years later, in 2010, Houk and Lee suggested considering male assignment for 46,XX completely virilized CAH patients (16). There is a trend that male assignment is becoming more accepted, and in severely virilized cases, even recommended (8, 16–18). An increasing number of recent case reports indicate that individuals with severe virilization, who were raised as male in a supportive environment, often demonstrate satisfactory social and sexual functioning, as well as overall positive outcomes in adulthood (16, 17, 19).

We identified 63 patients with a confirmed diagnosis of CAH, 46,XX karyotype and an initial male sex assignment in the literature (16, 19–36) (**Supplementary Table 1**). Prader staging at the diagnosis was available in 59 cases: 26 (26/59, 44%) presented with Prader stage V genitalia, another 25 with Prader stage IV genitalia (42%), one with Prader stage III-IV, and 7 (7/59, 12%) with Prader stage III genitalia. Outcome data were available for 50 of the 63 published cases. Male-to-female reassignment occurred in four cases, and all patients were satisfied with this decision (20, 26, 28, 29). Another three cases showed repeated gender reassignment (male to female and subsequently back to male gender) (16, 19). Since then, they have maintained a stable male gender identity. Overall, 43 patients (including those three individuals who underwent male-female-male reassignment) have a well-established male gender identity (43/50, 86%) (16, 19–24, 26, 28, 30–33, 36), while in three cases, the gender identity was unclear or reported as bigender (16, 24, 29). All patients with male sex assignment and male gender identity who married chose female partners according to the cited references where such information was available (16, 19, 20, 22). Five patients showed mild psychological issues, such as poor self-esteem, impaired social adjustment or depression (16, 24, 29) and one patient with stable male gender identity died by suicide after being informed of their female sex at 31 years of age (36). Follow-up was lost in one case (25), whereas in 12 cases follow-up is still ongoing (25, 27, 34, 35), but there is no evidence that any issues have arisen regarding male gender identity to date.

A majority of the patients were surgically treated (51/63, 81%). According to the data available at the time the case reports were published, overall, 43 patients underwent hysterectomy and 43 underwent oophorectomy. 22 patients had testicular prosthesis implantation, while 13 patients required chordee correction. Eight patients declined surgical treatment, while surgery is awaited in two cases (**Supplementary Table 1**).

Notably, one patient developed prostate carcinoma at 62 years of age after 50 years of testosterone therapy, emphasizing the importance of vigilance in patients with high levels of endogenous or exogenous testosterone (30).

## 4 Discussion

46,XX male is a rare condition in which genital virilization occurs during intrauterine life (1, 2). Until the 1950s, most of the patients were reared as male based on the appearance of the external genitalia (23). Later, as cytogenetic and molecular genetic techniques became more widely available, the age at diagnosis decreased and more patients were initially assigned as female or underwent a male to female reassignment (37).

The assignment of sex of rearing is a complex decision and the multidisciplinary team must consider several factors in collaboration with the patient and their family. Houk and Lee provided a thorough overview of these relevant factors for sex assignment: external genital development, psychosocial factors (family dynamics, social circumstances, cultural pressures), fetal CNS androgen exposure, anticipated quality of sexual function, fertility potential, and minimizing physical risk (gonadal cancer or urinary tract damage etc.) (8). Lee et al. found that familial and social support has a great impact on satisfactory self-esteem, male gender identity and masculinity (16). There are case reports in the literature where the male sex of rearing was chosen due to socioeconomic pressures, typically in male dominant cultures (19).

Shared decision-making among healthcare professionals, the patient, and the parents helps to ensure informed decisions and may reduce the fear of the “wrong” decision regarding sex assignment or other irreversible interventions (13, 38). Informing children about their condition in a way that matches their age and level of understanding is essential (14). Gender reassignment should be initiated solely by the patient (39). There is growing evidence that positive parental support, psycho-educative and psychological counseling of the families is needed for a good adjustment. It seems that the major predictor of adult gender identity outcome is the initial sex assignment and rearing (40, 41). In our cases, the male sex assignment happened in the newborn period and their gender was never questioned later. Presumably, this early decision and the consistency of the sex assignment may have had a beneficial effect on the development of their strong and stable gender identity.

The surgical interventions for 46,XX male patients may include salpingo-oophorectomy, hysterectomy, vaginal resection, hypospadias repair, chordee correction, and testicular prosthesis insertion, depending on the patient’s individual needs (19, 25). These procedures should aim to ensure the appropriate aesthetic appearance and functional outcome of the external genitalia in alignment with the individual’s gender identity, while also preventing potential complications such as urinary obstruction, urinary tract infections, voiding difficulties and erectile dysfunction.

Historically, early genital surgeries were often recommended for children with atypical genitalia; however, current recommendations emphasize the importance of obtaining the patient’s informed consent prior to any irreversible non-emergency surgical interventions. In the last decades, since the functional preservation policies emerged, the indication for any kind of surgical alteration has remained controversial. According to the latest Endo-ERN Expert Opinion from 2026 in accordance with current recommendations of the European Union, if no medical emergency exists, irreversible genital surgeries should be deferred until the individual can provide informed consent or should be performed under strict conditions until 12 years of age (42). As regulations vary significantly across countries, cultures, religions, the clinicians of the DSD medical team must remain mindful of these diverse perspectives, while preserving and prioritizing the patient’s autonomy is essential. Although delayed genital surgery is generally favored, individual considerations and the legal and ethical framework of the patient’s country must be accounted for in every case.

The positive aspect of male sex assignment is that external genital surgery is not needed during infancy and further decision regarding surgical intervention is possible later (43), although the exact timing of surgery remains debated (14). There is no clear evidence regarding the optimal timing, or specific indications for these procedures (44, 45). In cases where gonadectomy or hysterectomy is deferred, menstruation can be suppressed pharmacologically following the onset of puberty. Furthermore, if the ovaries remain intact, future fertility remains a viable option.

Our patients have complete male appearing genitalia with phenotypic male scrotal skin and urethral orifices on the tip of the glans. As the families of our patients were undoubtedly sure about the gender, oophorectomy was performed in early childhood, primarily to prevent breast development (**Table 1**). In our cases, these procedures (gonadectomy, hysterectomy) in minors were legally permissible, as these interventions were aligned with the sex recorded on the birth certificate and the parents as legal guardians provided informed consent after extensive counseling with the multidisciplinary medical team.

Overall, the main challenges and disadvantages associated with male sex assignment include infertility, the surgical removal of female reproductive organs, the need for testicular prosthesis and the possible need of testosterone treatment, which depends on the patient’s clinical needs and their satisfaction with endogenous testosterone levels.

Ovarian tissue cryopreservation may offer a promising option for fertility preservation in 46,XX CAH patients regardless of the sex assignment in the future (46) and in countries where surrogacy is legal, this could theoretically provide an opportunity for biological parenthood. Unfortunately, at that time, ovarian tissue or follicular cell cryopreservation was not available for our patients.

Genotypic females (46,XX) have a lower predicted adult height than genotypic males, which may lead to dissatisfaction or anxiety, as observed in our first patient. This aspect should also be discussed with the parents during the shared decision-making process regarding initial sex assignment, especially if the patient has genotypic male (46,XY) brother, as a greater height difference compared with his sibling is expected later in life.

Consistent with prior case reports, our experience indicate that male sex assignment may be an option for 46,XX CAH infants with severe virilization. Nevertheless, each case requires individualized consideration. Therefore, we emphasize the importance of careful evaluation of each individual living with DSD through open, multidisciplinary discussions that fully involve and inform the parents.

## 5 Conclusions

Patients born with atypical genitalia require individualized and holistic care with a patient-centered approach. While the decision regarding sex assignment for severely virilized 46,XX CAH patients necessitates careful consideration, our findings suggest that male sex assignment may represent a potential choice. In these cases not only strict medical factors, but also the patient’s and their family’s religious, cultural, and socioeconomic background, psychosocial needs, and the ethical and legal framework must be taken into account.

The shared decision-making process should be conducted among the parents and the multidisciplinary team over multiple sessions to ensure adequate time for delivering comprehensive information about the pathophysiology, treatment options and possible outcomes of CAH. If male sex assignment is ultimately chosen in a child with a 46,XX karyotype, the parents must be thoroughly informed and fully aware of the implications of fertility, the need for surgical interventions, potential height-related issues, and the possible requirement of testosterone treatment. Strong parental and family support appear to be crucial for the acceptance of the assigned gender and the achievement of a positive outcome.

Nevertheless, more comprehensive data, long-term follow-up are needed regarding long-term gender identity outcomes, psychological well-being, and particularly fertility-related distress in this unique population.

## Data Availability

The original contributions presented in the study are included in the article/**Supplementary Material**. Further inquiries can be directed to the corresponding author.
