# Supplementary material for: Male sex assignment in severely virilized 46,XX children with congenital adrenal hyperplasia
Source: Front Endocrinol (Lausanne). 2026 Mar 2;17:1765653. doi: 10.3389/fendo.2026.1765653 (PMC12989325; doi:10.3389/fendo.2026.1765653)
Supplement: Supplementary file 1 [file Table1.docx]

**Supplementary Table 1**

**46,XX CAH patients with initial male sex assignment – review of the literature**

| **Author and year of publication** | **Country** | **CAH diagnosis /mutation (if known)** | **Age at the diagnosis (years)** | **Diagnostic findings** | **Sex assignment** | **Gender identity** | **Surgical intervention (age/year if known)** | **Outcome and age at last follow-up (if known)** |
| --- | --- | --- | --- | --- | --- | --- | --- | --- |
| Madsen, 1963 (20) | Germany | CAH (not specified) | 35 | Prader V genitalia, cryptorchidism, hypertension, pubic and axillary hair developed at age 5, menstruation started at age 26 | male | female | left adrenalectomy 🡪 hypertension, shock 🡪 death | decided to transition to female gender at 35 years, (spontaneous menstruation at 26 years reinforced female gender identity) |
| Madsen, 1963 (20) | Germany | CAH (not specified) | 30 | Prader V genitalia, cryptorchidism, hypertension, menstruation (“hematuria”) started at age 22, (appendectomy at 23 years revealed ovaries and uterus) | male | male | (refused any operative procedure) | satisfied with male gender, married to a female, having regular sexual intercourse |
| Gillenwater et al. 1970 (21) | USA | CAH (not specified) | 4 (re-evaluated at 10 years) | marked masculinization at 4 years (facial, pubic, axillary hair and large penis), cryptorchidism, hypertension, intermittent “hematuria”, enlargement of the breasts at 7 years | male | male | hysterectomy, bilateral oophorectomy at 10 years of age (in 1959) | psychiatric evaluation at 10 years of age confirmed a definite male gender role |
| Money and Daléry, 1976 (22) | USA | CAH (not specified) | 12.17 | Prader V genitalia, cryptorchidism | male | male | hysterectomy, ovariectomy, testicular prosthesis implantation at 13 years (in 1970) | boyish play habits in childhood, female reassignment was advised but refused at 13 years, has fiancée at 18 years, but reports mild sexual dissatisfaction |
| Money and Daléry, 1976 (22) | USA | SW CAH | 0 | Prader V, cryptorchidism | male | male | hysterectomy, ovariectomy at 3.5 years (in 1954) | boyish play habits in childhood, married to a female, satisfied with male gender and sex life (at 24 years of age) |
| Money and Daléry, 1976 (22) | USA | CAH (not specified) | 7.5 | Prader V, cryptorchidism | male | male | hysterectomy, ovariectomy at 7.5 years (in 1956) | boyish play habits in childhood, married to a female, satisfied with male gender and sex life (at 26 years of age) |
| Chan-Cua et al. 1989 (23) | USA | SW CAH | 8.17 | adrenal crisis (vomiting, diarrhoea, electrolyte imbalance), bilateral cryptorchidism | male | male | ovaries and Müllerian structures removed, testicular prosthesis implantation (age NR) | strongly established male gender identity and behavior |
| Chan-Cua et al. 1989 (23) | USA | SV CAH | 4.34 | bilateral cryptorchidism, precocious puberty | male | male | hysterectomy, salpingo-oophorectomy, testicular prosthesis implantation at 6.34 years | strongly established male gender identity and behavior |
| Jones, 2004 [36] | USA | CAH (post mortem diagnosis) | 11 (disclosed at 31) | Prader IV genitalia, cryptorchidism, hypospadias | male | male | right salpingo-oophorectomy at 11 years (in 1915) | suicide at age 31, after being informed of female sex (cystoscopy revealed uterus and vagina) – previously satisfied with male gender and had a fiancée |
| Lee et al. 2010 (16) | USA | 21OH deficiency | 3 | Prader IV genitalia | male | male | hysterectomy, bilateral salpingo-oophorectomy, testicular prosthesis implantation (age NR) | sexual orientation to female, clear commitment to gender choice, family and social support reported (at 35 years of age) |
| Lee et al. 2010 (16) | USA | 21OH deficiency | 3-12 (only range given) | Prader IV genitalia | male (at birth)🡪 female (during infancy) 🡪 male (reassigned 2x) | male | feminizing genital surgery (female gender reassignment was decided in infancy) (age NR) | self-reassignment at 18 years, satisfied with his male gender, has a female partner, satisfactory sexual function (at 35 years of age) |
| Lee et al. 2010 (16) | USA | 21OH deficiency | 3-12 (only range given) | Prader V genitalia | male | male | hysterectomy, bilateral salpingo-oophorectomy, testicular prostheses (age NR) | satisfied with the male gender, married to a female (at 36 years of age) |
| Lee et al. 2010 (16) | USA | 21OH deficiency | 3-12 (only range given) | Prader IV genitalia | male | male | hysterectomy, bilateral salpingo-oophorectomy, testicular prostheses (age NR) | satisfied with the male gender, the male gender was never challenged, married to a female (at 45 years of age) |
| Lee et al. 2010 (16) | USA | 21OH deficiency | adulthood | Prader V genitalia | male | male | hysterectomy, bilateral salpingo-oophorectomy (age NR) | satisfied with the male gender, married to a female (at 46 years of age) |
| Lee et al. 2010 (16) | USA | 21OH deficiency | 3-12 (only range given) | Prader V genitalia | male | male | hysterectomy, bilateral salpingo-oophorectomy, testicular prostheses (age NR) | satisfied with the male gender, married to a female (at 47 years of age) |
| Lee et al. 2010 (16) | USA | 21OH deficiency | 3-12 (only range given) | Prader IV genitalia | male (at birth) 🡪 female (during infancy) 🡪 male (reassigned 2x) | male | feminizing genital surgery (during infancy) (age NR) | later self-reassigned male, he has a clear male gender identity at 49 years of age (“if genital repair were possible, he would like to have sexual relationship with women”) |
| Lee et al. 2010 (16) | USA | 21OH deficiency | 10 | Prader IV genitalia, “hematuria” (menstruation) | male | male | hysterectomy, bilateral salpingo-oophorectomy, testicular prostheses(age NR) | parental acceptance and support after the diagnosis; satisfied with male gender, married to a female (at 49 years of age) |
| Lee et al. 2010 (16) | USA | 21OH deficiency | 3 | Prader IV genitalia | male | male | hysterectomy, bilateral salpingo- oophorectomy, testicular prosthesis implantation (age NR) | equivocal gender guidance, lack of family support 🡪 poor self-esteem and social adjustment; married to a female partner (at 49 years of age) |
| Lee et al. 2010 (16) | USA | 21OH deficiency | 3-12 | Prader V genitalia | male | male | hysterectomy, bilateral salpingo-oophorectomy, testicular prosthesis implantation (age NR) | poor psychosocial adjustment, alcohol and drug abuse; married to a female partner (at 53 years of age) |
| Lee et al. 2010 (16) | USA | 21OH deficiency | 3 | Prader V genitalia | male | male | hysterectomy, bilateral salpingo-oophorectomy, testicular prosthesis implantation (age NR) | reasonable family support, but poor psychosocial adjustment; married to a female partner (at 57 years of age) |
| Lee et al. 2010 (16) | USA | 21OH deficiency | 3-12 | Prader IV genitalia | male | male | hysterectomy, bilateral salpingo-oophorectomy, testicular prosthesis implantation (age NR) | identified and lived as male (“He was always male”); priest, no sexual partner (at 69 years of age) |
| Sharma and Gupta, 2012 (24) | India | 21OH deficiency | 13 | Prader IV genitalia, hypospadias, cryptorchidism | male | male | hysterectomy, adnexectomy, chordee correction, urethroplasty, testicular prosthesis implantation (age NR) | stable male gender identity (at 29 years of age) |
| Sharma and Gupta, 2012 (24) | India | 21OH deficiency | 15 | Prader IV genitalia, hypospadias, cryptorchidism,breast development | male | male | hysterectomy, adnexectomy, chordee correction, urethroplasty, testicular prosthesis implantation (age NR) | satisfied with the male gender and the outcome of the surgical procedures (at 26 years of age) |
| Sharma and Gupta, 2012 (24) | India | 21OH deficiency | 13 | Prader IV genitalia, hypospadias, cryptorchidism,breast development | male | male | hysterectomy, adnexectomy, chordee correction, urethroplasty, testicular prosthesis implantation (age NR) | satisfied with the male gender and the outcome of the surgical procedures (at 23 years of age) |
| Sharma and Gupta, 2012 (24) | India | 21OH deficiency | 21 | Prader V genitalia,  gynecomastia, cryptorchidism, breast development | male | male | hysterectomy, adnexectomy, chordee correction, urethroplasty, testicular prosthesis implantation (age NR) | satisfied with the male gender and the outcome of the surgical procedures (at 30 years of age) |
| Sharma and Gupta, 2012 (24) | India | 21OH deficiency | 7 | Prader IV genitalia, severe hypospadias, cryptorchidism | male | male | hysterectomy, adnexectomy, chordee correction, urethroplasty, testicular prosthesis implantation (age NR) | satisfied with the male gender and the outcome of the surgical procedures (at 14 years of age) |
| Sharma and Gupta, 2012 (24) | India | 21OH deficiency | 14 | Prader IV genitalia, hypospadias, cryptorchidism, breast development | male | bigender | hysterectomy, adnexectomy, chordee correction, urethroplasty, testicular prosthesis implantation (age NR) | poor social adjustment, bigender mental makeup (at 20 years of age) |
| Sharma and Gupta, 2012 (24) | India | 21OH deficiency | 14.5 | Prader IV genitalia, hypospadias, cryptorchidism, breast development | male | male | hysterectomy, adnexectomy, chordee correction, urethroplasty, testicular prosthesis implantation (age NR) | satisfied with the male gender and the outcome of the surgical procedures (at 19 years of age) |
| Kirli et al. 2013 (25) | Turkey | 21OH deficiency | 5 | Prader V genitalia, cryptorchidism, penile chordee | male (decision made by gender assignment team and parents) | male | hysterectomy, bilateral salpingo-oophorectomy, vaginectomy, chordee release, urethral repair, fistula repair (age NR) | NA (follow up by team is ongoing) |
| Kirli et al. 2013 (25) | Turkey | 11OH deficiency | 2 | Prader V genitalia, cryptorchidism | male (decision made by gender assignment team and parents) | male | hysterectomy, bilateral salpingo-oophorectomy, vaginectomy (age NR) | NA (follow up ongoing) |
| Kirli et al. 2013 (25) | Turkey | 21OH deficiency | 0 | Prader V genitalia, penile chordee | male (family refused the offered female gender by the gender assignment team) | male |  | follow up lost |
| Kirli et al. 2013 (25) | Turkey | 21OH deficiency | 2 | Prader V genitalia, cryptorchidism | male (decision made by gender assignment team and parents) | male | hysterectomy, bilateral salpingo-oophorectomy, vaginectomy (age NR) | NA (follow-up ongoing) |
| Kirli et al. 2013 (25) | Turkey | 21OH deficiency | 6.5 | Prader IV genitalia, cryptorchidism | male (decision made by gender assignment team and parents) | male | hysterectomy, bilateral salpingo-oophorectomy, vaginectomy, chordee release, urethral repair, fistula repair (age NR) | NA (follow-up ongoing) |
| Kirli et al. 2013 (25) | Turkey | 11OH deficiency | 10 | Prader V genitalia, electrolyte imbalance | male (decision made by gender assignment team and parents) | male | hysterectomy, bilateral salpingo-oophorectomy, vaginectomy (age NR) | NA (follow-up ongoing) |
| Kirli et al. 2013 (25) | Turkey | 17OH deficiency | 6 | Prader IV genitalia, cryptorchidism | male (decision made by gender assignment team and parents) | male | hysterectomy, bilateral salpingo-oophorectomy, vaginectomy, urethral repair (age NR) | NA (follow-up ongoing) |
| Kirli et al. 2013 (25) | Turkey | 17OH deficiency | 2 | Prader V genitalia, cryptorchidism | male (decision made by gender assignment team and parents) | male | hysterectomy, bilateral salpingo-oophorectomy, vaginectomy (age NR) | NA (follow-up ongoing) |
| Kirli et al. 2013 (25) | Turkey | 21OH deficiency | 3 | Prader V genitalia, cryptorchidism | male (decision made by gender assignment team and parents) | male | hysterectomy, bilateral salpingo-oophorectomy, vaginectomy, eccentric circummeatal based glanuloplasty (age NR) | NA (follow-up ongoing) |
| Kirli et al. 2013 (25) | Turkey | 11OH deficiency | 3.5 | Prader IV genitalia, hyperpigmentation, | male (decision made by gender assignment team and parents) | male | hysterectomy, bilateral salpingo-oophorectomy, vaginectomy, chordee release, urethral repair, fistula repair (age NR) (and mastectomy at 15 years) | NA (follow-up ongoing) |
| Bin Abbas et al. 2014 (26) | Saudia Arabia | 11OH-deficiency  (novel mutation c.53_54insT) | 16 | Prader IV genitalia, hypospadias, cryptorchidism | male | male 🡪 female (parents reported that she behaved as a female since early childhood) | feminizing genitoplasty at 17 years | psychiatric evaluation confirmed female gender identity at 16 years, satisfied with female gender (at 26 years of age) |
| Bin Abbas et al. 2014 (26) | Saudia Arabia | 11OH-deficiency  (novel mutation c.53_54insT) | 14 | Prader V genitalia, cryptorchidism | male | male | hysterectomy, oophorectomy, testicular prosthesis implantation at 15 years (after >1 year of counselling and intensive psychiatric interview) | satisfied with male gender, normal erectile function and orgasm, but no relationship reported (at 24 years of age) |
| Bin Abbas et al. 2014 (26) | Saudia Arabia | 11OH-deficiency  (novel mutation c.53_54insT) | 10 | Prader V genitalia, cryptorchidism | male | male | hysterectomy, oophorectomy at 11 years | satisfied with male gender, normal erectile function and orgasm, but no relationship reported (at 20 years of age) |
| Nour and Pacaud, 2015 (27) | Pakistan | 11OH-deficiency | 3 | Prader III-IV genitalia, bilateral cryptorchidism, elevated 17-OHP (28 ng/mL, RR: <1 ng/mL), hypertension | male | male | hysterectomy, bilateral salpingo-oophorectomy at 3 years; hypospadias repair, chordee release, removal of breast tissue at 14 years | follow up ongoing |
| Gangaher et al., 2016 (28) | India | CAH (not specified) | 3.5 | Prader III genitalia | male | male | not performed | satisfied with the male gender (at 12 years of age) |
| Gangaher et al., 2016 (28) | India | SW CAH | 0 | Prader V genitalia, salt-wasting symptoms | male | male | not done (feminizing genitoplasty was refused) | satisfied with the male gender (at 8 years of age) |
| Gangaher et al., 2016 (28) | India | CAH (not specified) | 18 | Prader III genitalia, clitoromegaly | male | male 🡪 female | hysterectomy at 17 years; clitoroplasty, vaginoplasty at 30 years after gender re-assignment | successful gender reassignment at 30 years (2 years before the study), satisfied with female gender (at 32 years of age) |
| Razzaghy-Azar et al. 2017 (29) | Iran | 11OH-deficiency | 6 | Prader IV genitalia, cryptorchidism, Tanner stage 3 pseudo-puberty at 6 years | male | male (parents, the child and the surgeon refused sex reversal after the diagnosis) | hysterectomy, ovariectomy | unemployed, depressed, unsatisfied with her gender (at 36 years of age) |
| Razzaghy-Azar et al. 2017 (29) | Iran | 11OH-deficiency | 0 | Prader V genitalia, virilisation, cryptorchidism | male | male 🡪 female (at 12 years of age, after breast development and menstruation) | feminizing surgery at 12 years of age | satisfied with the female gender, married, has two children (at 34 years of age) |
| Wesselius et al. 2017 (30) | Nether-lands | 21OH-deficiency | 12 | Prader V genitalia | male | male | hysterectomy, adnexectomy (age NR) | 50 years of exogenous testosterone therapy resulted in prostate carcinoma at age 62 🡪 curative radiotherapy 🡪 PSA decreased, currently stable |
| Khattab et al. 2017 (19) | Pakistan | 21OH-deficiency | 3 | Prader IV genitalia | male | male | bilateral mastectomy, hysterectomy, vaginectomy, salpingo-oophorectomy, urogenital sinus and chordee repair, testicular prosthesis insertion at 13 years | satisfied with male gender role and behavior, married to a female, reported regular sexual intercourse (at 28 years) |
| Khattab et al. 2017 (19) | Brazil | 21OH-deficiency | 1.5-2 | Prader IV genitalia | male | male | chordee correction at 3 years, bilateral salpingo-oophorectomy, hypospadias repair, testicular prosthesis insertion at 8 years | satisfied and happy with male gender role and behavior, having girlfriend and regular sexual activity (at 27 years of age) |
| Khattab et al. 2017 (19) | Puerto Rico | 21OH-deficiency | 0.5 | Prader III genitalia | male (at age 17 months reassigned to female 🡪 at age 28 years reassigned to male) | male | vaginoplasty, clitoral resection at 2 years; clitorectomy at 12 years; vaginal dilation and vaginoplasty at 17 years; bilateral salpingo-oophorectomy at hysterectomy at 28 years | satisfied and happy with the male gender role and behavior, and married to a female (at 29 years of age) |
| Apóstolos et al. 2018 (31) | Brazil | 21OH- deficiency | 1.67 | Prader IV genitalia, phallus size 4 cm, cryptorchidism, hypospadias, | male (female gender was recommended after the diagnosis, but the parents did not accept it) | male | hysterectomy, oophorectomy, hypospadias correction, testicular prosthesis implantation at 8 years | satisfied with the male gender; normal erectile function, libido, orgasms, and has a girlfriend (at 27 years of age) |
| Apóstolos et al. 2018 (31) | Brazil | 21OH- deficiency | 12 | Prader V genitalia, phallus size 6 cm, “hematuria” (menstruation) --uterus and ovaries were found) | male (medical team and parents agreed to maintain male gender after the dg) | male | hysterectomy, bilateral oophorectomy at 12 years  (refused medication) | satisfied with male gender; normal erectile function, libido, orgasms, and has a girlfriend (at 27 years of age) |
| Apóstolos et al. 2018 (31) | Brazil | 21OH- deficiency | 5 | Prader IV genitalia, phallus size 5 cm, cryptorchidism, hypospadias | male (medical team and parents agreed to maintain male gender after the diagnosis) | male | hypospadias correction at 6 years, hysterectomy and oophorectomy at 10 years | satisfied with the male gender; normal erectile function, libido, orgasms, but no relationship reported (at 18 years of age) |
| Apóstolos et al. 2018 (31) | Brazil | 21OH- deficiency | 1.34 | Prader IV genitalia, phallus size 4 cm, cryptorchidism, hypospadias, | male (female gender was recommended after the dg, but the parents did not accept it) | male | hypospadias correction at 9 years, hysterectomy and oophorectomy at 12 years | satisfied with the male gender, normal erectile function, libido, orgasms, but never had sexual relationship (at 14 years of age) |
| Utari et al. 2022 (32) | Indonesia | 11OH-deficiency | 13 | Prader V genitalia, low cortisol levels, hypertension | male (decision made by the father at the age of 7 - based on his observations) | male | untreated * | satisfied with the male gender, “happy living as a male” (at 15 years of age) |
| Utari et al. 2022 (32) | Indonesia | 11OH-deficienty | 6 | Prader V genitalia, low cortisol levels, hypertension | male (decision was made by the father after birth) | male | untreated * | satisfied with the male gender (at 8 years of age) |
| Adriaansen et al. 2024 (33) | Indonesia | 21OH-deficiency (c.293-13A/C>G) | 15.6 | Prader III genitalia | male | male | untreated * | satisfied with the male gender |
| Adriaansen et al. 2024 (33) | Indonesia | SV CAH  CYP21A2 (c.293-13A/C>G) | 32.6 | Prader IV genitalia | male | male | untreated * | satisfied with the male gender |
| Adriaansen et al. 2024 (33) | Indonesia | 21OH-deficiency  CYP21A2 (c.586C>T) | 10.1 | Prader III genitalia, salt wasting crisis | male | male | untreated * | satisfied with the male gender |
| Adriaansen et al. 2024 (33) | Indonesia | SW CAH  CYP11B1 (c.(?_-107)  (447+)del) | 15.3 | Prader III genitalia, salt wasting crisis | male | male | untreated * | satisfied with the male gender |
| Bapir R et al. 2025 (34) | Iraq | CAH (not specified) | 10 | ambiguous genitalia, cryptorchidism, hypospadias,  two ovaries, no uterus | male (at birth) 🡪 delayed to allow gender choice after the diagnosis | gender and genital ambiguity management were delayed until the patient can make an informed decision | unilateral gonadectomy to preserve fertility (if reassigned female) | follow-up ongoing |
| Hamaichat et al. 2025 (35) | Morocco | CYP11B1 | 2 | Prader V genitalia, cryptorchidism, Tanner stage 3 pubic hair at 7 years, Tanner stage 4 breast development at 11 years | male | male | feminization surgery was declined by the parents at 7 years 🡪 follow up lost until 11 years 🡪 re-evaluation 🡪 planned masculinization procedure | follow-up ongoing |

Terminology in Supplementary Table 1 is used according to the cited reference.

NR= not reported in the cited reference

untreated * = Surgical intervention and glucocorticoid treatment was refused by the patient/parents due to the decline in endogenous adrenal androgen production, despite the risk for adrenal crisis.
